# Clinical Parameters, Subgingival Microbiota, and MMP-8 Expression in Dental Implants and Homologous Teeth: Insights into Peri-Implant and Periodontal Health

**DOI:** 10.1590/0103-644020266921

**Published:** 2026-05-01

**Authors:** Carla Dias Matos, Maria Cândida Dourado Pacheco, Marcílio Alves Ferraz, João Gabriel Silva Souza, Fernanda Faot, Karolina Ladeira Felisberto, Jamil Awad Shibli, Rodrigo Villamarim Soares, Elton Gonçalves Zenóbio

**Affiliations:** 1 Dental Implant Program, Department of Dentistry, Pontific Catholic University of Minas Gerais (PUC Minas), 30535-901, Belo Horizonte, Brazil; 2 Department of Periodontology, Dental Research Division, Guarulhos University, 07043-070, Guarulhos, SP, Brazil; 3 Faculdade Israelita de Ciências da Saúde Albert Einstein, Hospital Israelita Albert Einstein, São Paulo, Brazil; 4 Department of Restorative Dentistry - School of Dentistry, Federal University of Pelotas, Pelotas, RS, Brazil; 5 Department of Oral Medicine, Infection, and Immunity, Harvard School of Dental Medicine, Boston, Massachusetts, USA; 6 Department of Oral Health Sciences, Periodontology, KU Leuven & Dentistry, University Hospitals Leuven, Leuven, Belgium

**Keywords:** dental implants, clinical parameters, subgingival biofilm, crevicular fluid, MMP-8

## Abstract

This study aimed to evaluate and compare clinical parameters, subgingival microbiota composition, and matrix metalloproteinase-8 (MMP-8) expression between dental implants and homologous teeth under healthy conditions, across distinct implant platform designs. A total of 47 patients contributed 79 implants and 79 homologous teeth. Clinical parameters, including probing depth (PD) and dimensions of keratinized mucosa (HKM, TKM, and WKM), were assessed. Subgingival biofilm samples underwent analysis by checkerboard DNA-DNA hybridization. Peri-implant and gingival crevicular fluid were collected for MMP-8 quantification. Results: Implants showed significantly increased PD and reduced keratinized mucosa dimensions compared with teeth. While overall microbial loads were comparable, the microbiological profiles differed: *Actinomyces naeslundii I, Actinomyces oris, Prevotella intermedia*, and *Treponema denticola* were detected at higher levels in teeth. Conversely, *Prevotella nigrescens*, *Aggregatibacter actinomycetemcomitans A + B*, and *Selenomonas noxia* were more prevalent in implants. High levels of *Aggregatibacter actinomycetemcomitans A + B* and *Selenomonas noxia* were observed in the External Hexagon (EH) group. Importantly, MMP-8 expression was approximately 29% higher in implants than in teeth (p<0.05), regardless of the implant platform design. MMP-8 levels correlated exclusively with crevicular fluid volume, but not with other clinical parameters. Dental implants differ from homologous teeth, presenting increased PD, reduced keratinized mucosa height (HKM), and distinct microbial compositions. Although total bacterial levels were similar, implants showed elevated MMP-8 levels compared with teeth, independent of implant platform, suggesting distinct biochemical activity even in clinically healthy sites.

## Resumo

Este estudo teve como objetivo avaliar e comparar parâmetros clínicos, a composição da microbiota subgengival e a expressão da metaloproteinase de matriz-8 (MMP-8) entre implantes dentários e dentes homólogos, em condições de saúde e considerando distintos *designs* de plataforma de implantes. Materiais e Métodos**:** Um total de 47 pacientes contribuiu com 79 implantes e 79 dentes homólogos. Parâmetros clínicos, incluindo profundidade de sondagem (PS) e dimensões da mucosa ceratinizada (HKM, TKM, e WKM), foram avaliados. Amostras de biofilme subgengival foram submetidas à análise por hibridização DNA-DNA em *checkerboard*. O fluido crevicular peri-implantar e gengival foi coletado para quantificação de MMP-8. Resultados**:** Os implantes apresentaram um aumento significativo na PS e redução nas dimensões da mucosa ceratinizada em comparação com os dentes. Embora as cargas microbianas totais fossem comparáveis, os perfis microbiológicos diferiram: *Actinomyces naeslundii I, Actinomyces oris, Prevotella intermedia* e *Treponema denticola* foram detectados em níveis mais altos nos dentes. Inversamente, *Prevotella nigrescens, Aggregatibacter actinomycetemcomitans A + B* e *Selenomonas noxia* foram mais prevalentes nos implantes. Altos níveis de *Aggregatibacter actinomycetemcomitans A + B* e *Selenomonas noxia* foram observados no grupo de Hexágono Externo (HE). É importante ressaltar que a expressão de MMP-8 foi aproximadamente 29% mais alta em implantes do que em dentes (p<0.05), independentemente do *design* da plataforma do implante. Os níveis de MMP-8 correlacionaram-se exclusivamente com o volume do fluido crevicular, mas não com outros parâmetros clínicos. Conclusões**:** Implantes dentários diferem dos dentes homólogos, apresentando maior PS, menor altura da mucosa ceratinizada (HKM) e distintas composições microbianas. Embora os níveis totais de bactérias tenham sido semelhantes, os implantes apresentaram níveis elevados de MMP-8 em comparação com os dentes, independentemente da plataforma do implante, sugerindo uma atividade bioquímica distinta mesmo em sítios clinicamente saudáveis

**Figure:**
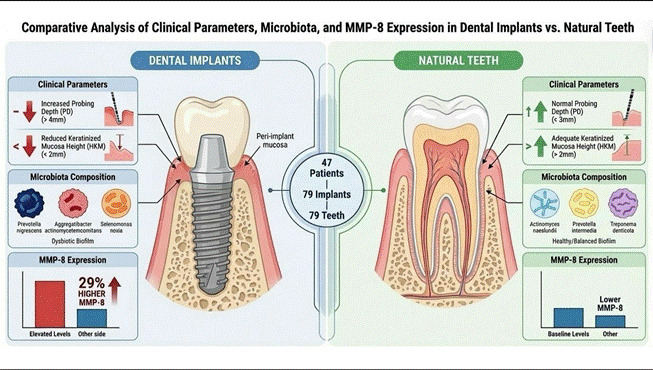

## Introduction

Dental implants have been considered the primary treatment for oral rehabilitation due to tooth loss. Nonetheless, once exposed to the oral environment, such as the tooth surface, implants are also exposed to microbial adhesion and accumulation, which often trigger implant-related infections ^1^. Implant-affected sites are characterized by inflammation in the surrounding mucosa and subsequent progressive loss of supporting bone ^2^. Although periodontitis and peri-implantitis share some similarities in terms of etiological factors (biofilm) and clinical signs of disease, previous evidence has suggested marked differences in biofilm biomass levels, microbiological shifts, and even higher clinical signs of inflammation at tooth sites compared with implants ^3^. Surface and microenvironment properties modulate biological processes, pathogenesis, and host response ^3^ ^,^ ^1^. Previous evidence has widely explored the biological and molecular events occurring during the inflammation process for tooth and dental implants, comparing both surfaces ^4^

Thus, biofilms act as a chemical-physical "stress" factor for surrounding tissues, activating an intense inflammatory reaction, leading to degradation of the extracellular matrix and alveolar bone resorption ^1^. Alongside the host response, the subgingival microbiota plays a pivotal role in the onset and progression of these diseases ^5^. The specific composition of the biofilm can modulate the inflammatory response and influence clinical outcomes. ^6^ ^,^ ^7^. Variations in the microbiota between implants and natural teeth, as well as among different implant connection designs, may provide critical insights for both the prevention and management of such pathologies.

Among the different factors acting during the inflammatory process, the proteolytic enzyme matrix metalloproteinase (MMP-8) has been suggested as a critical role in activating periodontal/peri-implant soft and hard tissue destruction ^8^. The MMPs are enzymes responsible for tissue degradation and repair. An increase in the MMP-8 expression and activity in different pathological conditions, which can lead to tissue destruction, has been previously reported ^9^. MMP-8 initiates the degradation of type I collagen, the most common collagen found in periodontal and peri-implant tissues. 

Moreover, this enzyme is the main interstitial collagenase present in the crevicular fluid of individuals with periodontitis and peri-implantitis ^10^. Interestingly, a previous systematic review identified a significant increase in MMP-8 levels at disease sites in periodontitis and peri-implantitis, which correlated with clinical signs of disease ^11^. Moreover, MMP-8 levels were reduced after disease treatment ^11^. Therefore, MMP-8 may be a potential diagnostic, predictive, and preventive biomarker for periodontal and peri-implant infections ^11^. However, previous evidence has focused mainly on comparing disease sites on the tooth and the implant. Although the last evidence evaluated MMP-8 levels at healthy implant sites ^12^, the correlation between clinical conditions of teeth and implants and implant design needs to be considered. A better understanding of how molecular factors affect the pathogenesis of peri-implant and periodontal diseases could lead to improved new therapeutic approaches. 

The intricate interplay among the host, the implant, and the microbial environment underscores the importance of an integrated approach that encompasses molecular and microbiological perspectives alongside clinical parameters. Within this context, the present study sought to evaluate and compare clinical outcomes, the composition of the subgingival microbiota, and the expression levels of matrix metalloproteinase-8 (MMP-8) between dental implants and their homologous teeth, both under healthy conditions and across distinct clinical statuses. Moreover, by examining the influence of different implant platform designs, this investigation aims to provide a more nuanced and comprehensive understanding of peri-implant and periodontal biology, thereby contributing to strategies for improved diagnosis, prevention, and management.

## Materials and methods

### Sample selection

The present study was approved by the Pontific Catholic University of Minas Gerais - PUC Minas (protocol #CAAE 03313512.0.0000.5137). Initially, 200 patients were screened. The inclusion criteria were based on the health status of teeth and implants, based on clinical evaluation. In addition, only implants loaded (under occlusal function) for at least six months were included. Individuals were excluded from this study if they were smokers, diabetics, immunosuppressed, osteoporotic, pregnant, HIV positive, had active periodontitis or peri-implantitis ^13^, or diagnosed with severe systemic alterations or had taken antibiotics and/or anti-inflammatory drugs within the past three months.

### Clinical evaluation

Periodontal parameters were assessed to diagnose clinically healthy implants and teeth without mucositis, gingivitis, peri-implantitis, or periodontitis. The following clinical parameters were considered: probing depth (PD), bleeding on probing, width of keratinized mucosa (WKM), thickness of keratinized mucosa (TKM), volume of crevicular fluid (VCF), implant-exposed threads, mobility, and intra-oral x-rays.

The PD was obtained using a UNC-15 periodontal probe (Hu-Friedy®, Chicago, IL, USA) in the central-vestibular and central-lingual sites. This probe was rotated mesially and distally around teeth and implants, and the deeper measurement at each site was recorded. Measurements > 4mm excluded the teeth and implants. Bleeding on probing was evaluated at the same sites dichotomously (present or absent); when present, it led to the exclusion of the implant and the teeth. The WKM was measured using a UNC-15 periodontal probe, recording the distance between the gingival margin and the mucogingival junction in the central buccal region of the implant or homologous tooth. The TKM ^14^ was measured in triplicate in the central buccal portion of the implant or teeth using a Periodontal Caliper® (Digimess, São Paulo, SP, Brazil). The examiners were trained and calibrated. Kappa index (K) was calculated to analyze the agreement, which showed good to excellent values (k= 0.79-1.0).

### Collection of subgingival biofilm samples

After removal of the supragingival biofilm with sterile gauze, the collection site was isolated with a cotton roll and carefully dried with compressed air. The samples were obtained from the deepest perimplant/periodontal site with a sterile type 11-12 Mini-Five Gracey curette (Hu-Friedy®, Chicago, IL, USA) positioned at the most apical portion of the site and in a single apical-coronal scraping motion. The samples were immediately deposited into tubes containing 0.15 mL of TE (10 mM Tris-HCl, 1 mM EDTA, pH 7.6). To each tube, 100 mL of 0.5 M NaOH was added. These tubes were then stored at -20° C until being sent to the Research Laboratory in Dentistry for analysis using the checkerboard DNA/DNA hybridization technique, including 40 bacterial species, as described by Shibli et al ^13^.

### Collection of peri-implant crevicular fluid (picf) and gingival crevicular fluid (gcf)

The clinically visible biofilm was removed, and the collection sites were isolated with cotton pellets. The gingival tissue was delicately dried with an air blast, and the PICF and GCF were collected using strips of absorbent paper filters (Periopaper®; Oralflow, New York, NY, USA). The paper strips were gently introduced into the sulcus until any resistance was eliminated, and they were maintained for 30 seconds. Samples visibly contaminated by blood were discarded, and the sites reevaluated during a later appointment. The collected fluid was evaluated using a Periotron® 8000 (Oralflow, Plainview, New York, NY, USA) immediately following collections and, in an attempt to reach the highest level of precision, a polynomial regression of the order of 4 was used to calculate the obtained volumes, as previously described by ^15^. These paper strips were placed in Eppendorf tubes and stored at -80°C. 

### Evaluation of the mmp-8 expression levels

To determine the MMP-8 concentration in the PICF and GCF samples, 300 μL of PBS containing 0.05% bovine serum albumin was added to the tubes containing absorbent paper strips. The tubes were shaken and left at rest for 40 minutes at room temperature. Next, the tubes were centrifuged at 6,000 RPM for 5 minutes, and the strips were removed. The samples were processed using ELISA kits (Human MMP-8 DuoSet, R&D Systems, Minneapolis, MN, USA) to quantify the MMP-8, following the manufacturer's instructions.

### Statistical analysis

The normality of the data was evaluated using the Kolmogorov-Smirnov or Shapiro-Wilk tests. The comparison between the two independent samples was carried out by applying the Mann-Whitney U test, and comparisons between two paired samples were performed using the Wilcoxon or Student t-test. The correlations between bacterial and MMP-8 levels and the periodontal/peri-implant parameters were tested using the Spearman rank correlation coefficient. The rates were compared using the Chi-squared test, Fisher's exact test, or McNemar's test. The significance level adopted in all analyses was 5% (α = 0.05). IBM SPSS Statistics for Windows ^16^ software was used.

## Results

### Periodontal and peri-implant parameter

The demographic characterization of included patients is presented in Box 1. For clinical parameters, the PD was higher for dental implants compared to teeth (p<0.05) (Table 1). However, the WKM found was significantly higher for teeth than for dental implants (p<0.05) (Table 1). According to implant platforms, EH and IH, dental implants showed similar clinical parameters.

**Box 1:**
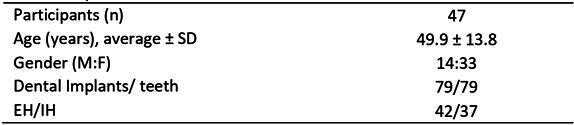
Sample Characterization

SD - standard deviation; EH: external hexagon implant; IH: internal hexagon

**Table 1:** Peri-implant and periodontal clinical parameters

Variable	Implants X Homologous teeth				Implant platforms (EH X IH)			
	Dental Implants	Teeth	p	EH	IH	p
PD (mm)^*^	2.00 ± 1.50	1.50 ± 1.00	< 0.001^†^	2.00 ± 0.50	2.00 ± 1.50	0.939
WKM (mm)^*^	2.00 ± 3.00	3.00 ± 3.00	< 0.001^†^	2.00 ± 3.00	2.00 ± 3.00	0.918
TKM (mm)^**^	0.88 ± 0.33	0.89 ± 0.30	0.817^‡^	0.86 ± 0.33	0.88 ± 0.52	0.721
VCF (µL)^*^	0.26 ± 0.24	0.25 ± 0.00	0.079^†^	0.26 ± 0.33	0.30 ± 0.23	0.965

PD - probing depth; WKM - width of keratinized mucosa; TKM - thickness of keratinized mucosa; VCF - volume of crevicular fluid; EH: external hexagon implant; IH: internal hexagon implant.

^*^ Values expressed in median ± IIQ; ^**^ Values expressed in average ± SD; ^†^ Wilcoxon test; ^‡^ Paired sample Student t test.

### Microbiological findings

Microbiological data from the peri-implant and periodontal biofilm samples are shown in Table 2. All 40 evaluated bacterial species were detected in the implant and in the homologous tooth samples. Five bacterial species differed significantly between the implants and homologous teeth: two from the *Actinomyces* group (*Actinomyces naeslundii I* and *Actinomyces oris*), two from the orange complex *(Prevotella intermedia* and *Prevotella nigrescens*), and *Treponema denticola* (red complex). Among the species that differed significantly, *A. naeslundii I, A. oris, P. intermedia, and T. denticola* were detected at much higher levels in the homologous tooth samples. *At the same time, P. Nigrescens* was detected in much higher levels in the implant samples. No significant differences were observed between the implant and homologous teeth groups in terms of total microorganism levels, combined levels of the six complexes, the *Actinomyces* group, or other species groups. Analyzing the peri-implant biofilm according to the EH and IH implants, all 40 evaluated bacterial species were detected in the EH implant group. However, only 38 species were detected in the IH implant group; the two species not detected were *Aggregatibacter actinomycetemcomitans A + B* and *Propionibacterium acnes I + II*. Only two bacterial species, *A. actinomycetemcomitans A + B* (green complex) and *Selenomonas noxia* (other species group), differed significantly between the EH and IH implant groups; both were detected at significantly higher levels in the EH implant group. A contingency analysis revealed that, of the 40 bacterial species evaluated, only *Selenomonas noxia* was detected at a significantly higher rate between the groups: (22.9%) in the EH implant group and (3.3%) in the IH implant group. 

The correlations between microorganism levels and clinical parameters in patients with implants and homologous teeth are presented in Table 3. Eleven bacterial species in the evaluated implants (*A. naeslundii I, C. gingivalis, F. nuc ss polymorphum, F. nuc ss vincentii, F. periodonticum, M. micra, P. nigrescens, T. forsythia, T. denticola, G. morbillorum, and N. mucosa*), three complexes (actinomyces, yellow, and red), the other species group, and the total microorganism level were also correlated with PD, HKM, or TKM. For homologous teeth, only seven evaluated species (*S. intermedius, F. nuc ss nucleatum, M. micra, P. nigrescens, T. denticola, E. saburreum, and S. anginosus*) and the red complex were correlated with HKM.

Table 3 also presents the correlations between the levels of specific microorganisms and the clinical parameters for the EH and IH implant groups. In the EH group, no significant correlations with PD were observed. However, several microorganisms showed significant negative correlations with HKM, including *A. naeslundii I* (r = -0.38, p = 0.024), *P. nigrescens* (r = -0.41, p = 0.015), *T. denticola* (r = -0.38, p = 0.026), *G. morbillorum* (r = -0.37, p = 0.027), *and Actinomyces spp*. (r = -0.34, p = 0.045), and the Red complex (r = -0.36, p = 0.034). No significant associations were identified for TKM in this group. In the IH group, a significant negative correlation was found between *A. israelii* and PD (r = -0.44, p = 0.015). Regarding HKM, negative associations were observed with *C. gingivalis* (r = -0.42, p = 0.021) and *G. morbillorum* (r = -0.46, p = 0.011). For TKM, several microorganisms showed significant negative correlations, including *F. periodonticum* (r = -0.40, p = 0.028), P. nigrescens (r = -0.50, p = 0.005), *G. morbillorum* (r = -0.46, p = 0.010), N. mucosa (r = -0.65, p < 0.001), and other species (r = -0.37, p = 0.044).

### MMP-8 findings

**Figure 1:**
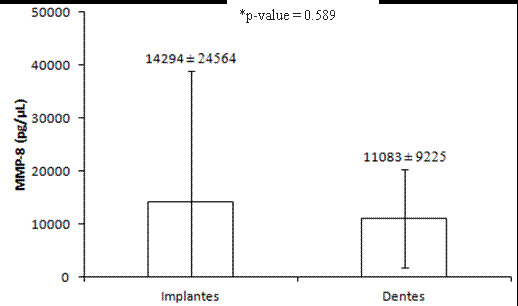
MMP-8 levels (pg/uL) in the peri-implant and periodontal crevicular fluid. MMP-8: Matrix Metalloproteinase-8.^*^ Wilcoxon test. Values are expressed as median ± IIQ.

**Figure 2:**
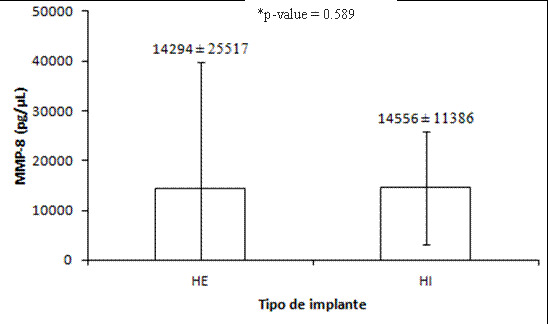
MMP-8 levels(pg/uL) in the peri-implant crevicular fluid. MMP-8: Matrix Metalloproteinase-8; EH: external hexagon implant; IH: internal hexagon implant. Values are expressed as median ± IIQ.^*^ Wilcoxon test.

The MMP-8 concentration in healthy peri-implant and periodontal crevicular fluid showed a higher concentration for implants (p<0.05) (Figure 1). The MMP-8 expression in the implants was approximately 29% higher than that found for teeth. According to the correlation between MMP-8 levels and clinical parameters, only a negative, significant (p<0.05) correlation was found between MMP-8 and the VCF for both substrates (Table 4). In addition, there was no difference in MMP-8 concentration in the crevicular fluid between the categories of WKM and TKM for both substrates (Table 5). According to implant platforms, they did not affect MMP-8 concentration in crevicular fluid (Figure 2), and only one negative and significant correlation was found between MMP-8 concentration and VCF (p<0.05) (Table 4). 

**Table 2:** Determination of microorganism levels (x10^5^) in the biofilm samples from implant, tooth, EH implants, and IH implants (Mean±SD).

Complex	Species	Implants X Homologous		P value*	Implant platforms (EH X IH)		P value*
		Implants	Teeth		EH	IH	
Actinomyces	*A. gerencseriae*	0.55±1.34	1.49±2.96	0.117	0.75±1.59	0.32±0.95	0.511
	*A. israelii*	3.82±12.45	4.72±12.50	0.117	2.56±3.11	5.30±18.07	0.935
	*A. naeslundii I*	0.13±0.64	1.50±2.96	0.001*	0.17±0.84	0.09±0.25	0.636
	*A. oris*	0.71±1.86	1.89±3.42	0.012*	0.91±2.25	0.49±1.27	0.904
Purple	*A. odontolyticus*	0.25±0.89	0.76±1.72	0.103	0.42±1.19	0.06±0.18	0.477
	*V. parvula*	3.76±17.43	3.84±17.42	0.580	3.87±16.98	3.62±18.23	0.456
Yellow	*S. gordonii*	0.20±0.88	0.37±1.50	0.618	0.22±0.86	0.18±0.91	0.363
	*S. intermedius*	0.42±1.83	0.22±0.89	0.692	0.48±1.86	0.35±1.82	0.583
	*S. mitis*	0.46±1.61	0.37±1.50	0.696	0.81±2.14	0.05±0.18	0.288
	*S. oralis*	0.27±0.90	0.28±1.38	0.385	0.41±1.19	0.11±0.30	0.242
	*S. sanguinis*	0.37±1.21	0.51±1.70	0.744	0.39±1.19	0.35±1.27	0.412
Green	*A. actinomycetemcomitans A + B*	0.01±0.03	0.01±0.03	1.000	0.01±0.04	0.00±0.00	0.033
	*C. gingivalis*	0.32±1.07	0.70±1.87	0.194	0.41±1.19	0.22±0.92	0.865
	*C. ochracea*	0.01±0.03	0.25±1.38	0.235	0.01±0.04	0.01±0.03	0.603
	*C. sputigena*	1.23±2.54	1.24±2.82	0.652	1.43±2.47	0.98±2.63	0.530
	*E. corrodens*	0.08±0.27	0.30±1.39	0.449	0.09±0.28	0.07±0.25	0.430
Orange	*C. gracilis*	0.67±1.65	0.51±1.61	0.827	0.89±1.90	0.42±1.27	0.882
	*C. rectus*	0.08±0.62	0.04±0.17	0.564	0.01±0.02	0.17±0.91	0.503
	*C. showae*	0.08±0.62	0.06±0.24	0.567	0.01±0.03	0.17±0.91	0.546
	*E. nodatum*	2.78±12.50	4.87±17.35	0.145	1.42±3.01	4.36±18.16	0.261
	*F. nuc ss nucleatum*	1.00±2.56	1.45±2.64	0.306	0.81±2.13	1.22±3.00	0.317
	*F. nuc ss polymorphum*	0.42±1.22	0.56±1.46	0.809	0.43±1.19	0.41±1.27	0.973
	*F. nuc ss vincentii*	6.80±24.12	5.72±21.07	0.717	6.44±23.47	7.22±25.25	0.177
	*F. periodonticum*	0.21±1.25	0.32±1.07	0.179	0.10±0.28	0.35±1.82	0.940
	*M. micra*	3.68±17.41	3.69±17.41	0.959	3.73±16.95	3.61±18.23	0.688
	*P. intermédia*	0.96±2.63	1.57±12.40	0.014*	0.91±2.56	1.02±2.75	0.313
	*P. nigrescens*	1.44±2.98	0.48±1.92	0.024*	1.75±3.18	1.08±2.74	0.861
	*S. constellatus*	0.32±1.07	0.14±0.65	0.351	0.38±1.19	0.25±0.93	0.950
Red	*T. forsythia*	0.28±1.39	0.44±1.61	0.163	0.21±0.87	0.37±1.83	0.304
	*P. gingivalis*	0.32±1.39	0.15±0.66	0.448	0.41±1.70	0.21±0.92	0.220
	*T. denticola*	2.01±2.33	3.18±3.38	0.008*	2.03±2.34	1.98±2.36	0.939
Other species	*E. saburreum*	0.10±0.63	0.17±0.67	0.307	0.18±0.86	<0.01±0.02	0.072
	*G. morbillorum*	0.38±1.22	0.54±1.45	0.330	0.64±1.61	0.09±0.25	0.639
	*L. buccalis*	0.46±1.34	0.83±2.20	0.303	0.53±1.42	0.37±1.27	0.276
	*N. mucosa*	0.98±2.24	1.78±3.18	0.059	1.01±2.23	0.95±2.28	0.905
	*P. melaninogenica*	1.01±2.12	0.99±2.23	0.954	1.27±2.41	0.70±1.72	0.760
	*P. acnes I + II*	<0.01±0.02	0.01±0.02	0.317	0.01±0.02	0.00±0.00	0.187
	*S. noxia*	0.12±0.64	0.14±0.65	0.606	0.21±0.87	<0.01±0.02	0.023*
	*S. anginosus*	0.56±2.18	0.34±1.50	0.714	0.59±2.35	0.52±2.01	0.964
	*T. socranskii*	1.88±2.68	5.56±17.29	0.807	2.28±2.86	1.43 ± 2.43	0.151

SD: Standard deviation; ^*^ Wilcoxon test.

**Table 3:** Correlations between microorganism levels and clinical parameters in implant and homologous teeth samples

Implant variables	PD		HKM		TKM	
	r_spearman_	p-value	r_spearman_	p-value	r_spearman_	p-value
*A. naeslundii I*	0.02	NS	- 0.36	0.003	- 0.16	NS
*C. gingivalis*	0.19	NS	- 0.25	0.045	- 0.12	NS
*F. nuc ss polymorphum*	0.26	0.039	- 0.26	0.035	- 0.14	NS
*F. nuc ss vincentii*	0.28	0.022	- 0.20	NS	0.02	NS
*F. periodonticum*	0.04	NS	- 0. 23	NS	- 0.25	0.042
*M. micra*	0.25	0.048	- 0.17	NS	- 0.20	NS
*P. nigrescens*	0.23	NS	- 0.28	0.022	- 0.26	0.040
*T. forsythia*	0.08	NS	- 0.26	0.039	- 0.13	NS
*T. denticola*	- 0.02	NS	- 0.33	0.008	- 0.08	NS
*G. morbillorum*	0.09	NS	- 0.41	0.001	- 0.18	NS
*N. mucosa*	0.06	NS	- 0.19	NS	- 0.43	<0.001
Actinomyces group	- 0.11	NS	- 0.26	0.036	- 0.16	NS
Yellow complex	0.17	NS	- 0.27	0.027	- 0.06	NS
Red complex	0.03	NS	- 0.33	0.008	- 0.09	NS
Other species	0.06	NS	- 0.27	0.032	- 0.28	0.026
Total	0.10	NS	- 0.26	0.038	- 0.22	NS
Homologous teeth variables						
*S. intermedius*	- 0.03	0.033	- 0.26	0.040	0.01	NS
*F. nuc ss nucleatum*	0.06	NS	- 0.32	0.010	0.10	NS
*M. micra*	0.08	NS	- 0.26	0.037	0.02	NS
*P. nigrescens*	0.02	NS	- 0.27	0.030	- 0.07	NS
*T. denticola*	- 0.21	NS	- 0.26	0.034	- 0.12	NS
*E. saburreum*	0.09	NS	- 0.31	0.013	0.07	NS
*S. anginosus*	0.03	NS	- 0.28	0.025	0.03	NS
Red complex	- 0.17	NS	- 0.25	0.044	- 0.13	NS
EH Implants variables						
*A. naeslundii I*	0.03	NS	- 0.38	0.024*	- 0.20	NS
*P. nigrescens*	0.23	NS	- 0.41	0.015*	- 0.02	NS
*T. denticola*	0.00	NS	- 0.38	0.026*	- 0.16	NS
*G. morbillorum*	0.17	NS	- 0.37	0.027*	0.05	NS
Actinomyces	0.07	NS	- 0.34	0.045*	- 0.15	NS
Red complex	0.07	NS	- 0.36	0.034*	- 0.16	NS
IH Implant variables						
*A. israelii*	- 0.44	0.015*	- 0.02	NS	- 0.22	NS
*C. gingivalis*	0.09	NS	- 0.42	0.021*	- 0.18	NS
*F. periodonticum*	- 0.08	NS	- 0.11	NS	- 0.40	0.028*
*P. nigrescens*	0.24	NS	- 0.10	NS	- 0.50	0.005*
*G. morbillorum*	0.02	NS	- 0.46	0.011*	- 0.46	0.010*
*N. mucosa*	- 0.16	NS	- 0.02	NS	- 0.65	< 0.001*
Other species	0.05	NS	- 0.23	NS	- 0.37	0.044*

PD: probing depth; HKM: height of keratinized mucosa; TKM: thickness of keratinized mucosa; NS: not significant.

**Table 4:** Correlation between MMP-8 expression levels and clinical parameters

Variables	Dental Implants	p-value	Teeth	p-value	EH	p-value	IH	p-value
PD	0.05	p = 0.700	-0.04	p = 0.741	0.08	p = 0.642	0.07	p = 0.742
WKM	-0.16	p = 0.232	0.17	p = 0.199	-0.18	p = 0.277	-0.04	p = 0.864
TKM	-0.05	p = 0.733	0.01	p = 0.968	0.07	p = 0.674	-0.24	p = 0.280
VCF	-0.59	p < 0.001	-0.71	p < 0.001	-0.62	p < 0.001	-0.50	p = 0.017

Spearman rank correlation coefficient; MMP-8: Matrix Metalloproteinase-8; PD - probing depth; WKM - width of keratinized mucosa; TKM - thickness of keratinized mucosa; VCF -the volume of crevicular fluid; EH: external hexagon implant; IH: internal hexagon implant.

**Table 5:** MMP-8 concentration according to the categories of WKM and TKM

Variable	Dental Implants	p-value^*^	Teeth	p*
WKM				
< 2 mm	15515 ± 23971	0.466	9725 ± 6892	0.256
≥ 2 mm	14268 ± 24924		12637 ± 9495	
TKM				
< 1 mm	14294 ± 24228	0.674	9725 ± 8594	.381
≥ 1 mm	13796 ± 29291		12754 ± 13968	

MMP-8: Matrix Metalloproteinase-8; Median ± IIQ; WKM - width of keratinized mucosa; TKM - thickness of keratinized mucosa. ^*^ Mann-Whitney U test.

## Discussion

This study analyzed clinical parameters, MMP-8 levels, and microbiological data from 40 bacterial samples collected from peri-implant sites, comparing them with those from homologous teeth. Additionally, all variables were examined in relation to their platform connections-external hexagon (EH) and internal hexagon (IH) implants. Our findings showed that periodontal and peri-implant sites, as well as EH and IH implants, showed similar clinical parameters (except for PD and WKM). The microbial differences observed between implant and homologous biofilms suggest distinct ecological dynamics in peri-implant and periodontal niches. The reduced levels of *A. naeslundii I, A. oris, P. intermedia,* and *T. denticola* at implant sites may indicate a less favorable environment for species typically associated with periodontal biofilms. Conversely, the higher prevalence of *P. nigrescens* in implant biofilms highlights a potential microbial shift toward species that may adapt more readily to the peri-implant environment. Overall, the data indicate that in the EH group, reduced keratinized mucosa height was consistently associated with higher levels of potentially pathogenic microorganisms. In contrast, in the IH group, both keratinized mucosa parameters (height and thickness) showed negative correlations with several species, particularly *N. mucosa* and *P. nigrescens*. These findings reinforce the notion that implants do not simply replicate the microbial profile of natural teeth but rather establish a unique biofilm composition, with implications for peri-implant health and disease susceptibility. 

In addition, since MMP-8 has been suggested as a biological biomarker for periodontal and implant infections and inflammation, its expression should be evaluated under normal, healthy conditions to correlate with clinical parameters. Our findings showed that crevicular fluid from healthy dental implants exhibited higher MMP-8 expression than the tooth substrate. Therefore, even under healthy conditions, dental implants show a higher level of MMP-8 than the tooth site, and this difference was not affected by the implant platform. 

### Peri-implant and periodontal clinical outcomes

In this study, probing depth (PD) was statistically higher around implants than around teeth, whereas the width of keratinized mucosa (WKM) was significantly lower around implants. This outcome may be attributed to the relative fragility of peri-implant tissues, as previously described by Parpaiola et al. ^17^. When comparing implants to homologous teeth, higher PD values were consistently found around implants, as described in previous studies comparing implants and contralateral teeth ^18^ ^,^ ^19^ ^,^ ^20^. A likely explanation for this difference lies in the weaker adhesion mechanism between the implant and mucosa compared to that between the tooth and gingiva. However, no significant differences in PD between implants and teeth were also reported, suggesting that other factors, such as patient variability or clinical scenarios, may influence this parameter ^21^. Interestingly, PD values between different implant platforms were similar, indicating that variations in implant-abutment connection design were subjected to the same biological and mechanical challenges. Supporting this, a recent randomized controlled trial (RCT) by Rubianes-Porta et al. ^22^ found no significant differences in PD between EX, IH, and conical connections in molar and premolar regions during the first year of loading. Furthermore, the risk of developing biological or mechanical complications appeared comparable across these connection designs.

The role of keratinized mucosa width (WKM) and thickness (TKM) in maintaining periodontal and peri-implant health remains a topic of debate. While some authors have suggested that keratinized mucosa plays a critical role in maintaining peri-implant health ^23^ ^,^ ^24^ ^,^ ^25^, other studies have concluded that its presence is not essential for patients with good oral hygiene ^26^ ^,^ ^27^ ^,^ ^28^. However, our study revealed that the WKM of implants was statistically lower than that of contralateral teeth. Similar findings were reported by Chang and Wennström ^15^, and this difference in soft-tissue topography could be attributed to the inherent fragility of peri-implant tissues ^29^ and to differences in adhesion mechanisms between implants and the mucosa compared with teeth and gingiva ^30^. Interestingly, this discrepancy is not always observed, likely due to differences in study protocols, methodologies, or sample characteristics ^21^. In our study, WKM measurements were obtained using a periodontal caliper®, following the method described by Yared et al. ^14^. While our results align with some prior findings, differences in outcomes could be partially explained by variations in the measurement techniques or smaller sample sizes in earlier studies. 

Despite this, the clinical significance of WKM remains of interest. Previous studies, including those by Chung et al.^31^, have shown that the absence of adequate keratinized mucosa, while not directly associated with greater bone loss, can contribute to increased plaque accumulation, peri-implant inflammation, and discomfort during oral hygiene maintenance. Recent systematic reviews ^32^ ^,^ ^33^ have emphasized that a WKM of 2 mm or more is essential for maintaining peri-implant and periodontal health. Additionally, Perussolo et al. ^34^ highlighted the protective role of keratinized mucosa in reducing plaque accumulation, tissue inflammation, and discomfort over time. Furthermore, a recent randomized controlled trial by Rubianes-Porta et al. ^22^ demonstrated that different implant-abutment connections (EX, IH, and conical) exhibited similar probing depths (PD) within the first year of loading, likely because they faced the same biological and mechanical challenges.

Differences in the volume of the PICF around implants and GCF, due to the presence of distinct structures in these tissues, have been described earlier ^35^, as has the finding that the volumes found correlate with peri-implant and gingival inflammation ^36^. No statistically significant differences could be observed in the VCF (Table 1). This finding may have resulted from the criteria adopted in the present study, which required implants and teeth to be clinically healthy.

### Microbiological outcomes

Previous studies have thoroughly examined and compared the clinical conditions and microbiota present around implants and contralateral teeth using the checkerboard DNA-DNA hybridization method ^18^ ^,^ ^37^ ^,^ ^20^. This highly specific technique avoids the limitations of traditional methods, such as dark-field microscopy, which can only identify bacterial morphology rather than specific species, and the culture method, which requires strict growth conditions for many bacterial species. In line with earlier investigations, our study found no significant difference in total bacterial levels between implants and homologous teeth; however, homologous teeth exhibited higher microbial counts, as reported in previous studies ^38^ ^,^ ^21^. Interestingly, studies have already identified significantly higher total bacterial levels in teeth ^37^ and around implants ^20^. Such conflicting results among studies could be explained by differences in sampling protocols, intraoral regions studied, and varying microbiological analysis techniques.

The ecological succession of bacterial biofilms provides an additional perspective on these findings. Early colonizers, such as those in the yellow (*e.g., Actinomyces*), green, and purple complexes, are not pathogenic and play a foundational role by creating a biofilm environment conducive to the proliferation of orange and red complexes. According to Socransky and Haffajee ^39^, the orange and red complexes harbor pathogens implicated in the progression of periodontal and peri-implant diseases. Therefore, while teeth may exhibit higher bacterial counts overall, this may reflect increased colonization by early, non-pathogenic complexes rather than by disease-related species. Differences in implant material and structure may also contribute, as implants often lack the native characteristics of enamel and dentin that favor bacterial adherence.

Further comparisons were made between the findings of this study and those of Cosyn et al. ^40^, particularly regarding the correlation between probing depth (PD) and microbiological levels around implants. Both studies confirmed bacterial involvement, as indicated by deeper PD measurements, although the specific bacterial species varied. For instance, this study identified significant associations with three species of bacteria (*F. nuc ss polymorphum, F. nuc ss vincentii, and Micromonas micra),* while Cosyn et al. reported associations with 4 different bacterial subsets *(Streptococcus oralis, Capnocytophaga gingivalis, Campylobacter showae, and Fusobacterium nucleatum sp. polymorphum*). Such discrepancies may stem from differences in sample selection, methodologies, or even variations in the microbial ecosystem between peri-implant and periodontal environments.

When analyzing current results on clinical parameters such as HKM (keratinized mucosa height) and TKM (keratinized mucosa thickness), our study identified a novel correlation yet to be explored in similar studies. For both implants and teeth, HKM showed a significant negative correlation with 12 and 8 bacterial species, respectively. Meanwhile, TKM was negatively correlated with bacterial levels in 4 species, but only in implant cases. These findings suggest that keratinized mucosa, particularly HKM, may act as a modulating factor in biofilm stability. Keratinized mucosa potentially enhances immunological resilience and mechanical protection, which could explain the observed correlations. Additionally, this protective role could be influenced by biomechanical factors, as adequate keratinized tissues enhance soft-tissue sealing around implants, making the peri-implant area less susceptible to bacterial invasion.

Our findings also highlighted significant differences in microbiota between external hexagon (EH) and internal hexagon (IH) implants. Notably, two bacterial species (*A. actinomycetemcomitans A+B and P. acnes I+II*) were not detected in IH implants, and *S. noxia* levels were significantly lower. Negative correlations were further noted between bacterial species and clinical parameters, with HKM being the predominant factor in EH implants. In contrast, TKM emerged as the key parameter in IH implants, reflecting structural and biomechanical differences induced by the implant-abutment connection. Specifically, 5 of 8 bacterial species showed negative correlations with TKM in IH implants, whereas HKM primarily influenced microbial load in EH implants.

These findings can be partially explained by the structural characteristics of the implant designs. The platform-switching design of IH implants, as extensively described in the literature, reduces bacterial contamination in the peri-implant area by moving the prosthetic gap away from the bone. This mechanism minimizes the inflammatory response and subsequent marginal bone loss. In contrast, EH implants are designed with a prosthetic system of the same diameter as the implant, creating a closer proximity to the bone margin. While these designs theoretically differ in their susceptibility to bacterial contamination, our clinical findings suggest that EH and IH implants demonstrated similar clinical behaviors in terms of bone loss and peri-implant health. The absence of significant differences between the two designs may be related to protocols promoting good oral hygiene among participants and to the short-term follow-up interval observed in the study.

### MMP-8 outcomes

Since MMP-8 has been widely recognized as a biological biomarker for periodontal and peri-implant infections and inflammation, its expression should also be assessed under normal and healthy conditions to better correlate with clinical parameters. Our findings demonstrated that although periodontal and peri-implant sites showed similar clinical parameters (except PD and WKM), crevicular fluid from healthy dental implants showed higher MMP-8 expression than that from the tooth substrate. This difference persisted even in the absence of active disease and was not affected by the implant platform.

The increased expression of MMP-8 in peri-implant sites, even under healthy conditions, aligns with earlier studies reporting elevated levels of this enzyme in peri-implant fluids, particularly in pathological states such as peri-implantitis ^10^. A plausible explanation for this observation relates to differences in tissue composition, as the higher proportion of collagen type II in the peri-implant mucosa compared to periodontal tissues may elevate MMP-8 levels. These findings suggest that physiological differences between teeth and implants, such as tissue type and structure, may inherently impact MMP-8 expression.

Interestingly, no correlation was found between clinical parameters (PD, WKM, and TKM) and MMP-8 expression in either group. However, a statistically significant negative correlation (p < 0.001) was identified between MMP-8 expression and crevicular fluid volume (VCF) in both implants and teeth. This may indicate that increased MMP-8 expression is associated with sites presenting lower VCF levels, even under healthy conditions. The lack of correlation between PD, WKM, or TKM and MMP-8 expression could be attributed to the healthy status of the evaluated sites, as reported in previous studies ^41^ ^,^ ^42^. 

Similarly, while prior studies ^43^ have suggested that changes in mucosal tissue might influence VCF production, our results did not show significant differences in the distribution rates of WKM and TKM, or in their association with MMP-8 expression across specific categories. The lack of significant differences in MMP-8 concentration across WKM and TKM categories suggests the complexity of peri-implant and periodontal biology. While WKM and TKM are commonly associated with better clinical stability-such as reduced inflammation, plaque accumulation, and probing depth-the findings indicate that MMP-8 expression, as an inflammatory biomarker, may not be directly influenced by soft tissue dimensions when implants and teeth are clinically healthy. This reinforces the idea that MMP-8 may serve as a biomarker more responsive to active inflammatory or diseased states, rather than to variations in baseline clinical parameters related to soft tissue morphology.

The influence of implant platform designs (EH vs. IH) on MMP-8 expression was also considered. No statistically significant differences were found between the two designs for clinical parameters (PD, WKM, and TKM) or MMP-8 expression. However, a notable negative correlation was observed between MMP-8 expression and VCF levels in both EH (p < 0.001) and IH implants (p = 0.017). Despite similar clinical parameters and MMP-8 levels observed across the two implant platforms, these findings warrant further investigation into the impact of specific implant designs on the underlying biological environment, particularly on inflammatory biomarker expression. Earlier studies ^20^ reported comparable clinical outcomes between Morse taper and IH implants, but correlations between clinical parameters and MMP-8 expression in EH and IH platforms have yet to be fully explored.

### Clinical implications

The findings of this study provide compelling insights into the structural, microbiological, and biochemical dynamics that define the intricate relationship between peri-implant and periodontal tissues. The observed mean difference of 1 mm in WKM between teeth and implants highlights the inherent structural vulnerability of peri-implant tissues, suggesting they may require additional clinical management to achieve long-term stability. While the necessity of keratinized mucosa for peri-implant health remains debated, an increasing body of evidence suggests its critical role in minimizing inflammation, plaque accumulation, and long-term discomfort, underscoring its undeniable relevance for both daily clinical practice and future research directions in implantology.

The exploration of microbiological profiles in this study underscores the complex interplay between biofilm composition and soft tissue characteristics. Correlations between bacterial species and parameters such as HKM and TKM reaffirm the hypothesis that soft tissue thickness and quality directly influence biofilm stability and, consequently, peri-implant and periodontal tissue health. Additionally, the differential impact of implant designs on bacterial colonization, particularly the advantages of platform switching, underscores the potential of structural modifications to enhance peri-implant health by reducing bacterial contamination.

Furthermore, elevated MMP-8 levels in peri-implant crevicular fluid, even under healthy clinical conditions, provide a new layer of understanding of the biochemical environment surrounding dental implants. While MMP-8 remains a valuable biomarker of inflammation and tissue remodeling, its higher expression in implants compared to teeth-regardless of implant platform-calls for caution in interpreting its diagnostic utility. These findings underline the physiological differences between peri-implant and periodontal tissues, urging clinicians and researchers to incorporate these variances into diagnostic protocols to improve accuracy and clinical decision-making. Importantly, while WKM and TKM remain key parameters for maintaining peri-implant health, the findings suggest that MMP-8 expression reflects broader tissue remodeling processes rather than being influenced by soft tissue dimensions under healthy conditions.

Finally, this study provides valuable insights into peri-implant and periodontal health, but it also has some limitations. Firstly, the sample size and cross-sectional design limit the generalizability of the findings and the ability to establish causation, underscoring the need for larger, longitudinal studies to assess changes over time. Additionally, the investigation was focused primarily on healthy sites, restricting its applicability to diseased states such as peri-implantitis or mucositis. While MMP-8 was evaluated as a key biomarker, the inclusion of additional inflammatory mediators and advanced microbiological techniques, such as 16S rRNA sequencing or metagenomics, could provide a more comprehensive understanding of the biofilm-host interactions. The study also lacked an assessment of mechanical or functional factors, such as occlusal loading and prosthetic misfit, which are known to influence peri-implant health.

Furthermore, the absence of patient-centered outcomes, such as discomfort, oral hygiene behaviors, or quality of life, limits the clinical relevance of the findings in routine practice. Future research should address these gaps by expanding the biomarker and microbiological panels, exploring diseased conditions, investigating the impact of mechanical forces, and incorporating patient-focused metrics. These efforts will enhance the understanding of peri-implant biology and support the development of improved preventive, diagnostic, and therapeutic strategies in implantology.

## Conclusion

Comparison of implants and homologous teeth showed increased PD and reduced HKM, while overall microorganism levels were similar. Five species differed significantly:*A. naeslundii I, A. oris, P. intermedia,* and*T. denticola*were higher in teeth, whereas*P. nigrescens*was higher in implants. EH-type implants exhibited elevated*A. actinomycetemcomitans A+B* and*S. noxia*. Healthy implants also had higher MMP-8 levels than teeth, independent of implant platforms. These results highlight the importance of considering microbial profiles and MMP-8 levels when evaluating peri-implant health.
